# Content Validation of the Self-Medication Scale and Trust in Online Resources: Deepening Digital Access to Health

**DOI:** 10.3390/nursrep14030141

**Published:** 2024-07-31

**Authors:** Eva Manuela Cotobal Calvo, Anna Bocchino, Concepción Mata-Pérez, Alberto Cruz-Barrientos, María Naranjo-Márquez, José Luis Palazón-Fernández

**Affiliations:** Salus Infirmorum Nursing Center, The University of Cádiz, 11003 Cádiz, Spain; evamanuela.cotobal@ca.uca.es (E.M.C.C.); conchi.mata@ca.uca.es (C.M.-P.); alberto.cruzba@ca.uca.es (A.C.-B.); marianaranjo@salusinfirmorumcadiz.com (M.N.-M.); jluis.palazon@uca.es (J.L.P.-F.)

**Keywords:** health professionals, self-medication, artificial intelligence, validation content

## Abstract

(1) Background: The rise of online health resources and AI has reshaped the diagnosis and treatment of disease, altering the relationship between patients and healthcare professionals and encouraging self-medication. However, questionnaires validated in the literature on self-medication seem to lack questions on the possible causes that induce such behaviors, including items that explore trust toward websites and artificial intelligence. The aim of this study is to design and validate the content of a questionnaire designed to assess self-medication in health professionals, delving into the underlying etiologies, the pharmacological categories involved and the degree of confidence placed in clinical assessments derived from AI tools. (2) Methods: Validation study of the content of an instrument consisting of two phases: 1. The content validation phase involved evaluation by a selected group of health experts, who, using a Likert-type scale, analyzed the clarity, coherence and relevance of the items. 2. Pilot study of health professionals who have indicated the practice of self-administration of medications. (3) Results: In the first phase of the study, the experts considered most of the questionnaire items to be clear, representative and consistent with the construct to be measured. In its second phase, the preliminary results of our pilot study suggest a significant trend toward self-medication among healthcare workers, along with a strong inclination to use online resources to search for health-related information. (4) Conclusions: The development of a validated instrument to measure the influence of the different causes that lead healthcare personnel to practice self-medication, guaranteeing validity and efficacy, allows us to establish strategies to reduce this growing problem.

## 1. Introduction

Access to online health information has undergone a progressive evolution that has revolutionized the way in which both healthcare professionals and the general population understand and manage healthcare [1]. Indeed, numerous technological advances have led to a proliferation of healthcare resources available on the Internet [2], making it easier to access online health information. Moreover, the emergence of artificial intelligence (AI) tools in healthcare has led to changes in the way diseases and health disorders are diagnosed and treated [3,4].

The accessibility of online health information guides patients and health professionals on symptomatology, treatments and health recommendations on the web, thus transforming the therapeutic alliance between patients and health professionals [5].

On the other hand, along with this ease of obtaining information, a trend toward self-medication has emerged among the general population, but also among health professionals [6].

Self-medication is defined by the WHO as “the selection and use of medicines by individuals to treat self-recognized diseases or symptoms” [7].

Self-medication is a public health problem that can cause adverse effects, such as masking of symptoms, delayed diagnosis of diseases and drug resistance [8].

Despite having healthcare training, healthcare professionals often resort to self-administration of medications and treatments based on information they find through websites, forums, blogs, social networking groups and AI [9,10,11].

Even social networking groups have become forums for discussion and consultation among professionals, where experiences are shared and second opinions are sought [12,13].

Inappropriate self-medication can lead to adverse consequences, iatrogenic diseases and masking of progressive diseases. Therefore, it represents an important problem that should be recognized and prevented, as it generates a great impact on public health. Specifically, this practice often bypasses the regulatory mechanisms that guarantee the quality of medical care [14,15]. Some of the consequences are a decrease in the efficacy of antibiotics due to the generation of resistant strains of bacteria, the appearance of side effects due to the use of drugs that are not indicated for the disease and, in some cases, direct damage to health due to the consumption of harmful products [15,16]. It is essential to raise awareness of the risks of self-medication and to promote comprehensive health education that includes pharmaco-safety and pharmaceutical care, especially considering that the COVID-19 pandemic and the consequent mobility restrictions have exacerbated the phenomenon of self-medication. This increase is attributed in part to the increased use of technologies and the difficulty in accessing medical care due to the saturation of health centers [17].

A study conducted during this period reveals that 36.3% of healthcare professionals resorted to self-medication. Despite possessing specialized knowledge in the health field, these professionals did not consistently exhibit behaviors that reflect safe health practices [18].

The exacerbation of self-medication during the COVID-19 pandemic, which has increased its prevalence in both the general population and among healthcare professionals, underscores the need to review and update the underlying causes and motivations for these practices. Various tools exist to analyze the causes and factors associated with self-medication [19,20,21,22,23], but these have not been adapted to recent advances in the technological context, particularly with regard to the influence of artificial intelligence (AI) on clinical decisions. Healthcare professionals’ reliance on AI-assisted clinical judgment could significantly influence their self-medication practices, so it is essential to update research methodologies to include these new paradigms.

In this context, the objective of the present study is to design and validate the content of a questionnaire designed to assess self-medication in health professionals, delving into the underlying etiologies, the pharmacological categories involved and the degree of confidence placed in clinical assessments derived from AI tools.

## 2. Materials and Methods

### 2.1. Phase 1. Instrument Design

An exhaustive literature review was carried out in Pubmed and Google Scholar databases, using the MeSH and DeCS “self medication”, “self-medication questionnaire” and “questionnaire”, combined by means of the Boolean operator AND, obtaining 13,817 results. After applying the filters “Full text”, “in the last 5 years”, “English” and “Spanish”, 322 results were obtained, which were analyzed by abstract, discarding those that did not directly study self-medication behavior.

#### 2.1.1. Selection of the Basic Questionnaires

Following the literature review, three questionnaires related to self-medication behavior were selected [21,22,23]. However, these three tools presented a number of barriers with respect to achieving the objectives set out in this study. In fact, none of the existing instruments were designed to assess the impact of artificial intelligence and social networks on self-medication practices, and all were answered by a specific population of healthcare professionals. Given the context and in line with the overall objective of the study, it was considered essential to analyze the level of trust placed in the clinical judgment exercised by artificial intelligence and social networks, extending it to a more diverse population of health professionals. In this way, we intend to address the methodological gap present in other existing tools.

The next step was to develop and validate a new questionnaire that integrates trust in the judgment of artificial intelligence and social networks. These two items are examined in relation to the self-medication decision and the confidence in the corresponding clinical assessment derived from them.

#### 2.1.2. Preparation of the Preliminary Vision

In this phase, a first version of the instrument was designed consisting of 3 sociodemographic variables and 14 items about prevalence, etiologies, pharmacological categories involved and degree of confidence in AI-based and/or social-network clinical assessments.

The research group named the instrument the Evaluation of Self-Medication and Confidence in Artificial Intelligence Assessment Scale (ESACIA). The items were chosen on the basis of the instruments already existing in the literature and the experience of the research team on the topic. The same research team identified the possible responses on the basis of already validated instruments.

### 2.2. Validation Process

#### 2.2.1. Phase 2. Validation of the Content of the Instrument

Content validity can be defined as the degree to which the items of an instrument are relevant and representative of the construct they are intended to measure. This can be done by means of a group of experts who evaluate the items of the instrument in terms of their clarity, coherence and relevance, using a Likert-type scale and following the criteria of Angleitner, John and Löhr [24], a procedure also recommended by Polit and Beck [25,26]. Clarity refers to the comprehensibility of the item with respect to syntax and semantics, coherence refers to the logical relationship of the item with the scale and relevance refers to the importance of including the item in the scale.

#### 2.2.2. Selection of the Expert Group

For the validation of this questionnaire, a sample of experts and health professionals was selected by means of purposive sampling [27]. The panel of experts was composed of different Spanish health professionals: nurses or nurse interns (EIR), midwives, physicians, psychologists, pharmacists and higher-level technicians in the health area from primary care centers or hospitals and having experience in self-medication.

The number of experts was selected according to the recommendations of Polit and Beck [25,26] in order to obtain useful estimates. A total of 30 experts were selected in this study.

#### 2.2.3. Validation Phase

After the explanation by the research team, this panel of experts assessed the clarity, coherence and relevance of each of the items of the questionnaire through a Google form questionnaire explaining the purpose of the development of this instrument. The sociodemographic data of the experts (sex, age and profession) were collected on the same form.

In this evaluation process, a 4-point Likert-type scale was used to measure clarity, coherence and relevance. The scale ranges from 1 (not at all clear, coherent or relevant) to 4 (very clear, coherent and relevant).

Finally, an open response space was added for suggestions or reformulation of items.

#### 2.2.4. Data Analysis

The statistical analysis of the data collected from the panel of experts included a descriptive analysis of the sociodemographic variables and the item and scale validity indexes (I-CVI and S-CVI). The I-CVI evaluates the proportion of experts who consider the content to be valid, that content being classified as excellent when it exceeds 0.78 [24,25]. As for the overall assessment of the instrument, the S-CVI is calculated by averaging the I-CVI values, with a value above 0.90 considered excellent [24].

#### 2.2.5. Collection of Feedback

In addition to the statistical analysis of the validity of the items, by means of the free question, the experts suggested inquiring about the types of sources most consulted prior to the practice of self-medication, as well as the causes that led participants to trust these sources.

#### 2.2.6. Revision of the Questionnaire

Once the pertinent modifications had been made by the committee of experts, the final version of the questionnaire was prepared. This integrated both the original items and those that were added later, thus ensuring an exhaustive and coherent understanding of the instrument as a whole.

Thus, the final version of the questionnaire is made up of 26 items, in turn divided into four areas:

(a) 3 items on sociodemographic data (age, sex, profession).
(b) 14 items related to the practice of self-medication (whether self-medication is practiced, reasons for self-medication, intensity, diseases treated, types of drugs, sources of information and drugs).
(c) 1 item on knowledge about medications (recommended dosage and posology, adverse effects, interactions).
(d) 8 items on attitudes toward self-medication.

### 2.3. Pilot Test

#### 2.3.1. Phase 3. Implementation of Pilot Study

After adjusting the questionnaire according to the recommendations obtained in the expert evaluation phase, a pilot study was carried out with 123 Spanish health professionals. Participants in the pilot study were recruited through telephone contacts and e-mails. Initially, e-mails were sent to the coordinators of various hospitals to distribute the information to other professionals. The questionnaire was administered online using Google Forms, which facilitated sample collection.

This initial study served to validate the new version of the instrument, consider possible contributions on the comprehension of the questions, verify the time needed to answer the instrument, etc. The selection of the sample for the pilot test was carried out using a non-probabilistic convenience sampling method.

#### 2.3.2. Data Analysis

The pilot sample was also used to calculate the reliability of the instrument as measured by Cronbach’s alpha coefficient [28] and to make an initial estimate of the prevalence of self-medication in health personnel, which served as the basis for calculating the sample size necessary for a study at the population level.

## 3. Results

**Phase 1.** *Literature review and instrument design*

After an exhaustive reading of these 46 articles, 38 of them were excluded for several reasons: they were not directly related to the research topic or they focused on study samples made up of non-health personnel. Finally, eight articles were considered relevant and adequate to contribute significantly to the research.

**Phase 2.** *Validation of the content of the instrument*

The content validity analysis was carried out with the participation of 30 experts. The sociodemographic characteristics of the expert group are shown in Table 1.

The results of the validation process of the self-medication and trust in online resources scale are described in Table 2, which shows the I-CVI of each item in relation to clarity, consistency and relevance. In all three aspects, all items obtained values above 0.78. An S-CVI value of 1 was obtained for clarity, 1 for coherence and 1 for relevance.

Most of the items obtained scores of 1 in terms of relevance, consistency and clarity.

The item that obtained the lowest I-CVI with respect to relevance, consistency and clarity was frequency of drug use through self-medication, which was reformulated. It should be noted that none of these items were excluded as their I-CVIs were considered adequate.

**Phase 3.** *Pilot test and statistical analysis*

The test was applied to a pilot sample of 123 people from the health sciences area to test the clarity and applicability/viability of the study tool and detect any difficulties that might arise during the application of the instrument, verify that the questionnaire recipients correctly understand the questions, see whether some open questions could be closed from the answer options provided by the respondents and verify the time required to answer the instrument. Based on the results obtained in the pilot sample, the necessary modifications were made to the final version of the questionnaire (changes in language, closure of items, order of some questions, etc.). The pilot sample was also used to calculate the reliability of the instrument, measured by Cronbach’s alpha coefficient at 0.78 (values above 0.70 are acceptable), and to make a first estimate of the prevalence of self-medication among health personnel, which served as the basis for calculating the sample size of the study at the population level. Table 3 shows the sociodemographic characteristics of the pilot study participants.

Regarding self-medication practices, a large majority of respondents (82%) indicated that they self-medicate. Within this group, the consumption of drugs by self-medication per week varies, with a frequency of one drug per week.

Regarding the search for health information on the Internet, a high percentage of the participants (74%) stated that they search for information related to health or pathologies via the Internet, artificial intelligence or social networks.

## 4. Discussion

With respect to the results obtained in the different phases of the study, the following should be highlighted:

1. The literature review showed a lack of specific instruments to assess the degree of trust placed in clinical assessments derived from AI tools. This methodological gap is probably due to the fact that theories concerning the role that AI plays in our lives are still unexplored due to the recent arrival of these new tools.

Moreover, despite the existence of different scales found in the literature [21,22,23], their specificity due to the samples used hinders their use in some research fields.

2. To validate the content of the instrument, the theoretical criteria mentioned in the literature [25,26] were applied through an expert judgment panel, followed by the calculation of the content validity indexes (I-CVI and S-CVI). The final instrument consisted of 23 items.

The results show that the ESACIA achieved excellent content validity, reflected in average values of 1 for clarity, relevance and coherence. Most of the items received outstanding scores, being considered clear, relevant and coherent with respect to the construct assessed. In addition, the consideration of the research team in including the other items suggested by the experts reflects, as in other research, the importance currently given to artificial intelligence specifically in the field of health sciences [29].

3. With respect to the pilot study, it should be noted that the most prevalent result was related to the search for health information on the Internet. Seventy-four percent of the participants stated that they searched for health-related information, diagnoses or pathologies via the Internet, artificial intelligence or social networks. These data highlight the importance and impact of information technologies on the health practices of healthcare personnel [30].

### Limitations

The use of an expert sample in this study entails several limitations in terms of subjectivity and generalizability of the results. The subjective nature of expert opinion may introduce bias into the study. In addition, the generalizability of the results to international populations may be limited, given that the questionnaire was developed considering the cultural characteristics of a single country (Spain). This may imply the need for future cross-cultural adaptations of certain items to different contexts. In addition, the high percentage of women in the study indicates that the sample is not gender-equitable. This limitation will be taken into account in future research.

## 5. Conclusions

The development of a validated instrument to measure the influence of the different causes that lead healthcare personnel to practice self-medication, guaranteeing validity and efficacy, makes it possible to establish strategies to reduce this growing problem. Preliminary results from our pilot study suggest a significant trend toward self-medication among healthcare personnel, along with a strong inclination to use online resources to search for health-related information. These trends highlight the need for further education and resources for healthcare workers on self-medication and the critical use of online health-related information. Future research should further explore the implications of these practices and how they may influence patient care and the health of healthcare workers themselves.

## Figures and Tables

**Table 1:** Sociodemographic characteristics of the experts.

Variables	n = 30
Gender	
Man	7 (23.3)
Woman	22 (73.3)
Other	1 (3.3)
Age	37.83 ± 9.10
Profession	
Nurse	11 (36.7)
Midwife	9 (30.0)
Physician	8 (26.7)
Psychologist	1 (3.3)

Qualitative variables are expressed as frequencies (%). Quantitative variables are expressed as mean ± sd.

**Table 2:** Item (I-CVI) and scale (S-CVI) content validity indexes.

Item	Clarity		Coherence		Relevance	
	I-CVI	Expert Agreeing	I-CVI	Expert Agreeing	I-CVI	Expert Agreeing
Item 1.	1.00	30/30	1.00	30/30	1.00	30/30
Item 2.	1.00	30/30	1.00	30/30	1.00	30/30
Item 3.	1.00	30/30	1.00	30/30	1.00	30/30
Item 4.	1.00	30/30	1.00	30/30	1.00	30/30
Item 5.	0.97	29/30	1.00	30/30	1.00	30/30
Item 6.	1.00	30/30	1.00	30/30	1.00	30/30
Item 7.	1.00	30/30	1.00	30/30	1.00	30/30
Item 8.	1.00	30/30	1.00	30/30	1.00	30/30
Item 9.	1.00	30/30	1.00	30/30	1.00	30/30
Item 10.	1.00	30/30	1.00	30/30	1.00	30/30
Item 11.	1.00	30/30	1.00	30/30	1.00	30/30
Item 12.	1.00	30/30	1.00	30/30	1.00	30/30
Item 13.	0.97	29/30	0.97	29/30	0.97	29/30
Item 14.	1.00	30/30	1.00	30/30	1.00	30/30
Item 15.	1.00	30/30	1.00	30/30	1.00	30/30
Item 16.	1.00	30/30	1.00	30/30	1.00	30/30
Item 17.	1.00	30/30	1.00	30/30	1.00	30/30
Item 18.	1.00	30/30	1.00	30/30	1.00	30/30
Item 19.	1.00	30/30	1.00	30/30	1.00	30/30
Item 20.	1.00	30/30	1.00	30/30	1.00	30/30
Item 21.	1.00	30/30	1.00	30/30	1.00	30/30
Item 22.	1.00	30/30	1.00	30/30	1.00	30/30
Item 23.	1.00	30/30	1.00	30/30	1.00	30/30
S-CVI *	1.00		1.00		1.00	

* S-CVI= average of the I-CVI.

**Table 3:** Sociodemographic characteristics of the pilot sample.

Variables	n = 123
Gender	
Woman	91 (74.0)
Man	30 (24.4)
Other	1 (0.8)
I prefer not to say	1 (0.8)
Age	45.76 ± 12.94
Profession	
Nurse	93 (75.6)
Nursing Student	8 (6.5)
Intermediate technician	5 (4.1)
Higher technician	1 (0.8)
Physician	5 (4.1)
Physiotherapist	2 (1.6)
Dentist	2 (1.6)
Psychologist	2 (1.6)
Biologist	1 (0.8)
Caretaker	1 (0.8)
Retired Nurse	1 (0.8)
Cleaner	1 (0.8)
Podiatrist	1 (0.8)

Qualitative variables are expressed as frequencies (%). Quantitative variables are expressed as mean ± sd.

## Data Availability

The data presented in this study are available on request from the corresponding author. The data are not publicly available due to privacy.

## Public Involvement Statement

There was no public involvement in any aspect of this research.

## Guidelines and Standards Statement

This manuscript was drafted according to the STROBE guidelines.
